# Gastric Schwannoma: A Case Report and Review of the Literature for Gastric Submucosal Masses Distinction

**DOI:** 10.1155/2018/1230285

**Published:** 2018-04-10

**Authors:** Behnam Sanei, Amirhosein Kefayat, Melika Samadi, Parvin Goli, Mohammad Hossein Sanei, Mahsa Khodadustan

**Affiliations:** ^1^Department of Surgery, Isfahan University of Medical Sciences, Isfahan, Iran; ^2^Cancer Prevention Research Center, Isfahan University of Medical Sciences, Isfahan, Iran; ^3^Department of Oncology, Seyed Al-Shohada Hospital, Isfahan University of Medical Sciences, Isfahan, Iran; ^4^Department of Pathology, Isfahan University of Medical Sciences, Isfahan, Iran; ^5^Department of Internal Medicine, Isfahan University of Medical Sciences, Isfahan, Iran

## Abstract

Schwannomas origin from Schwann cells sheath and generally are benign, slow-growing, and asymptomatic neoplasms which frequently appear in the head and neck. Although gastrointestinal schwannoma is really rare, the most affected organ in GI system is the stomach. Gastric schwannoma forms 0.2% of all gastric tumors. This neoplasm is always detected as a submucosal mass, the same as other gastrointestinal stromal tumors. Although these tumors have almost the same presentations, they are completely different at therapeutic options and prognoses. Hence, it is important to distinguish them apart and make an accurate diagnosis to optimize treatment outcomes. Herein, we report a case of 28-year-old woman with frequent vomiting and abdominal pain caused by 5 × 6 cm schwannoma in the antrum of the stomach. This is a rare case of gastric outlet obstruction due to a massive schwannoma. In addition, all other probable submucosal masses will be discussed at different aspects.

## 1. Introduction

Submucosal tumors (SMTs) include a wide spectrum of benign-to-malignant lesions. SMT employs to define the intramural neoplasms underneath the mucosa. The incidence of SMTs in the whole gastrointestinal (GI) tract is unknown. However, the stomach is frequently the most affected organ [1]. Gastric SMTs are divided into three major categories according to the ultrastructural characteristics and protein marker profiles including myogenic tumors, neurogenic tumors, and gastrointestinal stromal tumors (GISTs) [2]. Theses lesions are usually asymptomatic and incidentally diagnosed during various diagnostic procedures. When the physician encounters such lesions, it is necessary to distinguish them from each other for choosing the appropriate therapeutic option.

In this case report, a 28-year-old woman with a 5 × 6 cm mass in the antrum of the stomach will be presented. She was complaining of frequent vomiting (sometimes bloody) and abdominal pain. After the surgery, the complaints were relieved and histopathological and immunohistochemistry assessments revealed a gastric schwannoma. In addition, other SMTs, as differential diagnoses, will be discussed at different aspects.

## 2. Case Presentation

**Figure 1:**
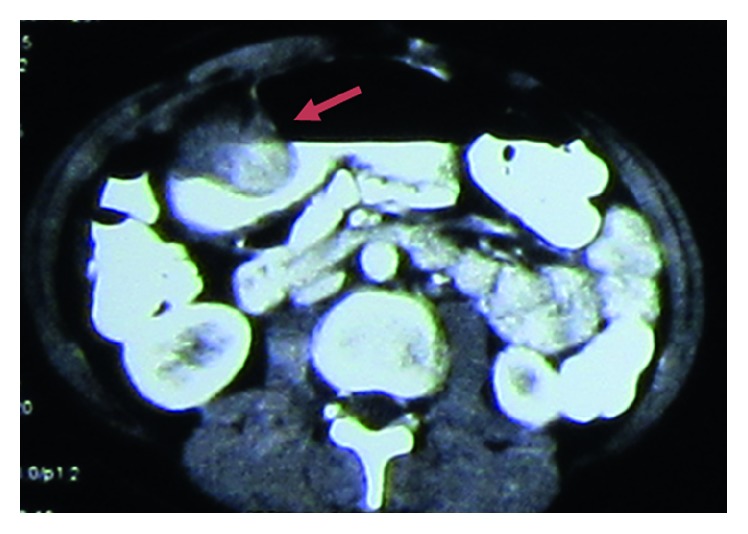
Abdominopelvic CT scan with IV and oral contrast. A 5 cm × 6 cm soft, hypodense mass along the anterior wall of the gastric antrum with subsequent pyloric stenosis and luminal obstruction with gastric dilatation in the body and fundus (the red arrow indicates the mass).

**Figure 2:**
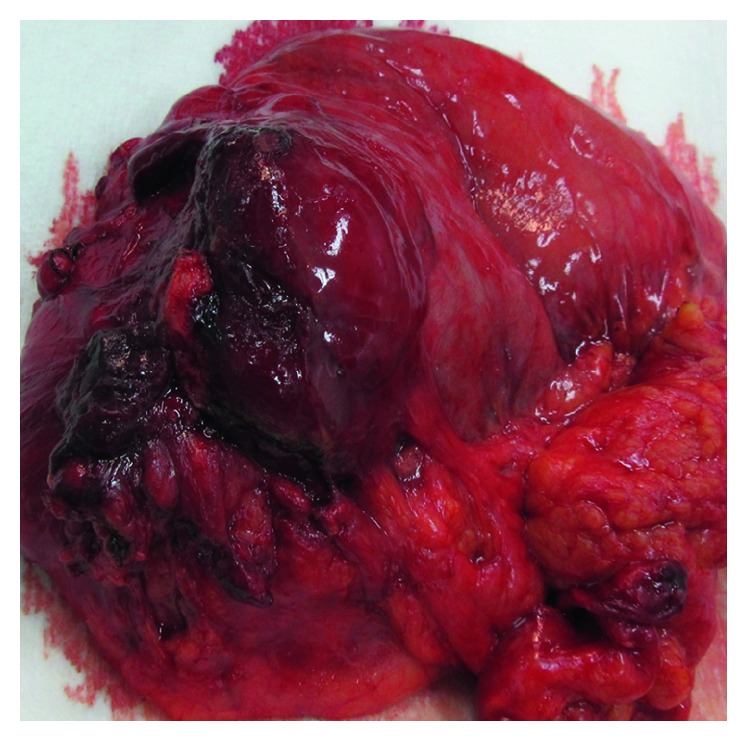
Resected gastric mass after surgery.

A 28-years-old woman was admitted to our emergency department due to abdominal pain, recurrent vomiting, and hematemesis. The patient had been suffering from abdominal pain for several weeks, and the symptoms gradually have worsened until the time of admission when she had severe anorexia and recurrent vomiting. She did not have any systemic and local complication, and the physical examinations did not reveal anything special. After the admission, the patient underwent diagnostic endoscopic study which showed a 5 cm × 6 cm antral submucosal mass with central umbilication. The mass was causing gastric outlet obstruction. Double contrast-enhanced abdominopelvic CT scan was performed and revealed a hypodense mass along the anterior wall of the gastric antrum with subsequent pyloric stenosis and luminal obstruction with gastric dilatation in the body and fundus (Figure 1). Possible GIST was suggested, and the patient was consulted for the surgery. The open surgery for the obstruction relief was done, and subtotal gastrectomy with Roux-en-Y gastrojejunostomy was successfully performed (Figure 2).

**Figure 3:**
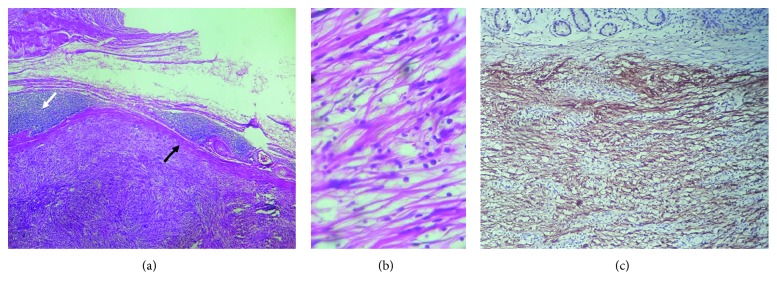
Histopathological and immunohistochemical examinations. (a) Wavy spindled cells separated by woven fibrotic bundles which encompassed by the capsule (black arrow) are apparent. Also, the tumor capsule is surrounded by lymphoid follicles (white arrow). (b) High-power field view. (c) S100 protein is apparent at tumoral parts with diffuse staining as a characteristic feature for schwannoma.

Pathological examinations were done, and characteristic histological findings for gastric schwannoma were detected (Figure 3). The tumoral capsule which encompassed wavy spindled cells separated by fibrotic bundles woven together in various directions was observed. In addition, lymphoid aggregations around the capsule were apparent. Surgical margins were free, and 3 reactive lymph nodes were seen. Subsequent immunohistochemical staining was performed, and the tumor cells were negative for CD117, CD34, KI67, actin, chromogranin, and desmin. However, they were positive for S100 protein diffusely at nucleus and cytoplasm. Based on histology and immunohistochemistry findings, the final diagnosis of gastric submucosal schwannoma was considered.

## 3. Discussion

The SMTs are divided into three major categories including myogenic tumors (leiomyomas or leiomyosarcomas), neurogenic tumors (schwannomas, granular cell tumors, and neurofibromas), and GISTs [3].

Gastrointestinal neurogenics derive from different components of nerve fibers, mostly autonomic components of Auerbach's plexus [4]. They consist 5% of nonepithelial gastric tumors and are mostly benign with 10% malignant transformation risk [5, 6]. Schwannoma, neurofibroma, and granular cell tumors are their subtypes. Schwannomas consist about 91% of the neurogenic tumors [7]. Neurofibroma is associated with the von Recklinghausen's disease. Therefore, the GI tract-related nerve sheath neoplasms mostly are detected at neurofibromatosis patients with apparent systemic manifestations like café au lait spots. Isolated GI neurofibroma without any associated systemic syndrome is an extremely rare clinical entity and forms 0.1% of all gastric neoplasms and frequently has been discovered in the small intestine [8]. Schwannomas arise from Schwann cells sheath. They are generally benign, slow-growing, and asymptomatic neoplasms which commonly occur in the head and neck. However, the GI involvement is really rare. The most common site for GI schwannomas is the stomach representing 0.2% of all gastric tumors, and malignant transformation risk is low [9–11]. Granular cell tumors (GCTs) are extremely rare neoplasm.

Myogenic tumors include leiomyomas and leiomyosarcomas which are rare and can be found anywhere along the GI tract. However, they are more common in the esophagus, stomach, and colon and arise in muscularis mucosae or muscularis propria [12].

GIST is the most common kind of submucosal mesenchymal neoplasms of the GI tract. The incidence of GISTs is 10–20 per million and the stomach is the most common location. Approximately 60% to 70% of GISTs arise in this organ and form 0.1%–3.0% of all GI malignant tumors [13, 14]. GISTs arise from Cajal cells which are the smooth muscles pacemaker cells in the GI tract. KIT gene mutation is the main factor for development of GISTs as 85% cases are positive for this mutation [15, 16]. Therefore, GIST's definition, diagnosis, and treatment are dependent on this mutation. Due to remarkable response of GISTs to the imatinib treatment as a molecular inhibitor of c-Kit, its accurate diagnosis from other gastrointestinal SMTs is vital [16].

Gastric SMTs frequently are so small and noninvasive to become symptomatic. According to a report by Perea and Gregory, abdominal pain is the most common symptom. GI bleeding, anemia, epigastric mass, nausea, and vomiting can also occur. Dyspeptic symptoms, anorexia, and epigastric tenderness are among rare symptoms [2, 17]. About 10–30% of GISTs are completely asymptomatic. Usually big GISTs, more than 6 cm in diameter, can cause symptoms, and the most common presentation is abdominal pain and/or GI bleeding [18, 19]. The upper GI bleeding has been named as the most common symptom for symptomatic schwannoma [20, 21].

It is obvious that clinical symptoms are nonspecific, and distinction of gastric submucosal masses with clinical presentation is approximately impossible. The standard procedures for establishing diagnoses in patients with GI tumors are endoscopic mucosal forceps biopsy. However, its false negative rate can be as high as 50%. Therefore, the endoscopic ultrasound (EUS) and EUS-guided biopsy (EUS-B) would be helpful as the most accurate procedures for definite diagnosis of GI tumors with negative endoscopic biopsies. EUS shows typical findings for some lesions, but in hypoechoic lesions, such as leiomyomas, GISTs, and schwannomas, EUS findings are not enough and EUS-Bs are needed. Due to deeper location than epithelium, biopsies cannot be taken from the subepithelial lesions by conventional endoscopic methods, and EUS-B should be employed [22, 23].

Also, imaging techniques are needed and computed tomography (CT) is a more common diagnostic tool for these cases. The CT scan presentation of GISTs depends on its size, aggressiveness, and the course of the disease. Primary GISTs are mostly large hypervascular lesions which exhibit as enhancing masses at contrast-enhanced CT scans with heterogeneous presentations due to necrosis, hemorrhage, or cystic degeneration [24, 25]. In comparison with GISTs, gastric schwannomas more frequently exhibit a homogeneous enhancement, exophytic, or mixed growth pattern with cystic degenerations. Due to overlapping properties at imaging, it is impossible to distinguish GIST and leiomyomas from each other with imaging modalities. Therefore, the final diagnosis is established by immunohistochemical examination [26–28]. Gastric neurofibromas at CT scan exhibit hypoattenuated mass lesion with usually well-defined borders. Tumor can be intramural or extramural, and central calcification is probable. Regardless of the origin, it will exhibit heterogeneous enhancement after contrast administration. Hemorrhage or necrosis occur at giant masses and will be detected as low-density component. These changes plus size and attenuation asymmetry can be predictive factors for malignant transformation [29, 30].

Although imaging techniques are helpful, histopathological analyses should be established to confirm the diagnosis [31]. The neurofibroma is usually described as nonencapsulated tumors consisting of Schwann cells, endoneurial fibroblasts, and perineurial-like cells in a context of mucinous matrix with wavy fibrotic collagenous bundles which are surrounded with mast cells infiltrations [32]. GI schwannomas are capsulated tumors consisting of spindle cells with prominent lymphoid aggregations which are characterized by Antoni A and Antoni B areas, and the absence of typical Verocay bodies [33, 34]. 70% of all GISTs contain spindle-shaped cell, and 20% are of epithelioid type at the histopathology. However, they also can be pleomorphic [35]. Leiomyomas, the same as the GIST, are of spindle-shaped cells. Therefore, their distinction just can be confirmed by immunohistochemistry examinations [36].

Immunohistochemical analysis should be done to approve the probable diagnoses in all SMTs. GISTs are mostly considered CD117-positive, generally CD34-positive, actin-positive, and S100-negative [19, 31, 37]. In addition, desmin staining can help for distinction between the GISTs with myogenic tumors. Desmin expression is negative at the GIST and positive for the myogenic tumors, while others are the same [38, 39]. Positive staining for S100 protein and vimentin and negative staining for smooth muscle actin, c-KIT, and CD34 supports the notion that the tumor is neurogenic. There are some evidences that schwannomas have a greater extent of S100 with a diffuse pattern expression in comparison with neurofibromas. It is obvious that relying on expression profile of these markers is not sufficient for distinction of these two subtypes of neurogenic tumors. Therefore, new studies introduce calretinin as a helpful diagnostic marker for neurogenic tumor distinguishing from each other [7].

In our case, the tumor cells were negative for CD117, CD34, and actin while were positive for S100. Therefore, GIST and myogenic tumors were excluded, and histological pattern of lymphoid aggregation plus capsule presence highly suggested schwannoma rather than neurofibroma.

This case was operated due to relief of obstruction symptoms with a probable diagnosis of GIST. In the course of surgery due to the presence of the 5 cm × 6 cm mass in the lesser curvature of gastric antrum and to obtain a 2 cm safety margin for the mass resection, subtotal gastrectomy was evitable. The lesser curvature masses resection causes vague nerve eradication and subsequent pylorus dysfunction. Therefore, for the lesser curvature masses, subtotal gastrectomy is the choice plan. In addition, subtotal gastrectomy is the treatment of choice for middle- and distal-third gastric neoplasm [40]. Therefore, the open surgery for subtotal gastrectomy and Roux-en-Y gastrojejunostomy was done. The definitive diagnosis of schwannoma was determined through postoperative pathological assessment.
